# Advancing prion diagnostics: full-length human E200K RT-QuIC substrate facilitates prion detection in tear fluid and improves sensitivity in cerebrospinal fluid

**DOI:** 10.1186/s40478-025-02212-8

**Published:** 2026-01-22

**Authors:** Susana Da Silva Correia, Matthias Schmitz, Peter Hermann, Stefan Goebel, Jaqueline Gerecke, Paul Lingor, Fabian Maass, Anna-Lisa Fischer, Sezgi Canaslan, Hasier Eraña, Joaquín Castilla, Angela Da Silva Correia, Inga Zerr

**Affiliations:** 1 National Reference Center for Transmissible Spongiform Encephalopathies, Department of Neurology, University Medical Center, Georg-August-University, Robert Koch Straße 40, 37075 Göttingen, Germany; 2 Department of Neurology, Klinikum rechts der Isar of the Technical University of Munich, Ismaninger Straße 22D, 81675 Munich, Germany; 3 Department of Neurology, University Medical Center, Robert Koch Straße 40, 37075 Göttingen, Germany; 4 Center for Cooperative Research in Biosciences (CIC BioGUNE), Basque Research and Technology Alliance (BRTA), Parque Tecnológico de Bizkaia, Edif. 801, 48160 Derio, Spain; 5 Centro de Investigación Biomédica en Red de Enfermedades infecciosas (CIBERINFEC), Carlos III National Health Institute, Av. Monforte de Lemos 3-5, 28029 Madrid, Spain; 6 ATLAS Molecular Pharma S. L., Parque Tecnológico de Bizkaia, Edif. 800, 48160 Derio, Spain; 7 IKERBASQUE, Basque Foundation for Science, Plaza Euskadi 5, 48009 Bilbao, Spain

**Keywords:** Diagnostic, Seeding assays, RT-QuIC, Prion diseases, Tear fluid, Prion protein

## Abstract

The real-time quaking-induced conversion (RT-QuIC) assay has revolutionized prion disease diagnosis by detecting amyloidogenic PrP conformers in different tissue types and body fluids. Recently, we achieved a breakthrough by detecting amyloidogenic PrP conformers in tear fluid (TF), a non-invasive biofluid, warranting further evaluation. We refined our RT-QuIC protocol to assess seeding conversion efficiency using various recombinant substrates, including full-length human (FL Hu), and mutant versions linked to genetic prion diseases, such as E200K and D178N, in both CSF and TF samples. Our study included patients with sporadic and familial prion diseases. FL Hu E200K showed the highest seeding efficiency in cerebrospinal fluid (CSF), with sensitivity increasing from 78 to 93% for symptomatic Creutzfeldt–Jakob disease (CJD) and from 19 to 75% for fatal familial insomnia (FFI) compared with the hamster-sheep substrate. For tear fluid, diagnostic sensitivity was 85% for sCJD and 64% for symptomatic genetic prion diseases, the assay was also able to detect amyloidogenic PrP in 57% of the healthy mutation carriers (HMC, persons at risk without symptoms). We validated the diagnostic accuracy of the TF RT-QuIC from our previous study (cohort 1) in a second independent study. Additionally, we confirmed that TF RT-QuIC requires FL Hu E200K substrates since hamster-sheep failed in detection of seeding activity in tears. Notably, only 1 out of 184 controls without prion disease tested positive. Studies comparing different disease stages (early vs. late) showed that later stages produced a stronger signal response. Our study demonstrates that the FL Hu E200K rec PrP substrate improves RT-QuIC sensitivity in CSF diagnostics and validates the reliable detection of seeding activity in TF in two cohorts as well as in HMC.

**Supplementary Information:**

The online version contains supplementary material available at 10.1186/s40478-025-02212-8.

## Introduction

Transmissible spongiform encephalopathies, or prion diseases, are characterized by the aggregation and accumulation of prion protein scrapie (PrP^Sc^) in brain tissue. Since the beginning of systematic clinical studies of sCJD, several paraclinical tests have been suggested [11, 38]. The development of aggregation assays, such as RT-QuIC, sets new standards in diagnosis by enabling the amplification and detection of PrP^Sc^, initially in CSF, later also in other non-brain tissues (olfactory mucosa and skin biopsies) from sCJD patients [1, 17, 20, 23] and in postmortem tissues [21, 26].

Since the first description, the test was modified with respect to the reaction procedure and substrate [9]. The research on different rec PrP substrates, such as bank vole, chimeric hamster-sheep, or human (wild type and including a *PRNP* mutation), aimed to improve diagnostic accuracy for atypical and genetic prion diseases [18, 25]. However, the sensitivity of the CSF RT-QuIC for atypical sCJD and some forms of genetic prion diseases, such as fatal familial insomnia (FFI) and Gerstmann–Sträussler–Scheinker syndrome (GSS), remained relatively low [12, 31]. Given the potential side effects of the CSF lumbar puncture, the search for easily accessible biomarker platform continues, addressing the needs of non-invasive sample collection procedure in longitudinal studies.

A recent breakthrough is the successful application of the RT-QuIC assay for diagnosing prion diseases using tear fluid (TF) samples of prion disease cases and healthy mutation carriers (TF and other biofluids) [22, 30, 32, 33]. This finding opens new perspectives to apply this test for the analysis of pre-clinical patient samples and/or follow-up studies to evaluate the efficiency of therapeutic intervention.

To further develop and validate this approach, we present a modified protocol utilizing FL Hu E200K recombinant PrP substrate for improved seeded amplification and detection of amyloidogenic PrP conformers in CSF and TF samples of patients with prion diseases and HMC. We addressed pre-analytical assay conditions, such as assay stability and the minimum required volume of TF, and we validated the diagnostic accuracy of TF RT-QuIC in an additional cohort. Moreover, we examined the effect of the disease stage on the RT-QuIC response.

## Methods

### Overall study scheme

The presented study on the RT-QuIC assay using FL Hu E200K rec PrP as substrate and its application in CSF and TF was performed in the following steps:

1. Modification and optimization of the RT-QuIC assay protocol for the analysis of CSF and TF: Comparative evaluation of different substrates (chimeric hamster-sheep, FL Hu, FL Hu D178N, and FL Hu E200K) under the same experimental conditions.
2. Evaluation of the test performance of the modified assays for prion disease diagnostic in CSF from sporadic and genetic prion diseases.
3. Analysis of the (pre)-analytical conditions of the TF RT-QuIC.
4. Determination of the overall diagnostic accuracy of the TF RT-QuIC and to validate the diagnostic accuracy of the TF RT-QuIC from our previous study (cohort 1) [30] in a new independent cohort 2 in a blinded manner regarding the patients specimens.

### Patients

Prion disease patients were recruited through the German National Reference Center for Transmissible Spongiform Encephalopathies. Control patients were enrolled as part of a prospective observational biomarker study. Diagnoses were established based on consensus criteria and *PRNP* mutation analyzes [11]. TF samples were collected during both early and late stages of the disease. Early-stage disease was defined as a state in which the clinical CJD criteria [11] were not fully met at the time of CSF or TF sampling. The control cohorts for CSF analysis consisted of patients diagnosed with non-neurodegenerative disorders, including psychiatric conditions, pain syndrome, vertigo, headache, and epilepsy. For the TF analysis, the control group included healthy donors and patients with non-prion diseases diagnosis, such as immune-mediated neurological disorders, Alzheimer’s disease, Parkinson’s diseases, neurovascular disease, and others.

TF samples from healthy mutation carriers (HMC, non symptomatic persons at risk) were collected from individuals with pathogenic *PRNP* variants confirmed through genetic testing, but exhibiting no neuropsychiatric signs of prion disease at the time of collection.

The E200K mutation carriers were collected in collaboration with the Spanish Foundation for prion diseases, under appropriate informed consent and the approval of the local Ethics Committee (CEIm-E). At the time of TF collection, HMC exhibited no neuropsychiatric signs indicative of prion disease. Over the course of up to 39 months of follow-up, none of the HMC developed prion disease.

A demographic overview of the patient cohort including codon 129 MV genotype and disease duration is shown in Table S1.

### Body fluid sampling and RT-QuIC protocols

CSF was collected according to standard procedures for diagnostic purposes. After diagnostic workup, the remaining CSF was aliquoted and stored at -80 °C without further modification until analysis. TF was collected from both eyes using Schirmer strips (Optitech, Allahabad, India) [30] under standard usage terms and extracted after storage at -80 °C [30]. CSF and TF RT-QuIC analysis was performed according to the modified protocol [30]. The development of the FL Hu E200K rec PrP substrate is described in Supplementary methods.

### Collection and pre-analytical handling of tear fluid

A 5 mm wide and 35 mm long strip was placed at the inferior eyelid margin in the outer corner for 8–10 min without topical anesthesia. The procedure corresponds to the standard quantification of tear production (so-called Schirmer test) in ophthalmology. According to the manufacturer’s manual, the test strips must not be applied to patients with corneal inflammation or corneal ulcer. None of these conditions were present in the study cohort. Samples were frozen < 30 min after sampling and stored at − 80 °C but not treated in any other way. Proteins were eluted from the strip (minimum 15 mm with liquid) by cutting the strips in 5 mm pieces, transferring them into a 1.5 mL Eppendorf cup and adding 50μL RT-QuIC reaction buffer. Afterwards, tubes were vortexed for 1 min followed by an incubation step of 30 min. Subsequently, strips were transferred to a 0.5 mL Eppendorf cup with a little hole in the bottom and placed on previous 1.5 mL Eppendorf cups containing solved proteins in the reaction buffer. To remove rest-liquid from the strips, we centrifuged the strip at 14,000 rpm (Eppendorf centrifuge 5810 R) at 4 °C for 10 min and collected the extracted tears. Samples from both eyes were pooled for analysis.

### RT-QuIC protocol

In each well, 85 μL of RT-QuIC buffer (162 mM phosphate buffer (pH 6.9), 170 mM sodium chloride, 1 mM ethylenediaminetetraacetic acid (EDTA), 10 μM thioflavin-T (Th-T) and 0.1 mg/mL recPrP) were seeded with 15 μL of CSF or extracted TF, respectively, adding up to a total volume of 100 μL. Analysis was carried out using a FLUOStar Omega plate reader (BMG LABTECH GmbH, Ortenberg, Germany).

Due to the lower seeding activity, TF RT-QuIC assay was run at 42 °C for 150 h (CSF: 80 h) for each substrate with intermittent shaking cycles (1 min double orbital shaking at the highest speed (600 rpm)) followed by 1 min incubation. Every 30 min, fluorescence signals were detected at 450 nm excitation and 480 nm emission.

Each patient sample was run in triplicates on the same plate. A positive signal response in the RT-QuIC was defined by a signal increase of ≥ 50% of relative fluorescence units (r.f.u.) from the assay’s baseline before 150 h passed. Test positivity was constituted when at least two out of three replicates showed a positive signal response. If one of three initial replicates was positive, three additional replicates were run, and the overall test was rated positive when at least three out of six replicates (≥ 50%) showed positive signal responses. These criteria were applied to CSF RT-QuIC before and validated in a large surveillance study [12].

While the procedure is generally considered acceptably safe under standard prion safety practices, it is recommended to adopt a cautious approach due to the potential generation of amyloid fibrils and aggregates. To minimize risks, it is suggested to perform pipetting in a biosafety cabinet if aerosol generation is possible, use sealed tubes to prevent contamination, minimize splash risk, and rigorously decontaminate surfaces and consumables with prion-appropriate protocols. Regarding the use of tears as a diagnostic sample, studies have shown that the PrP concentration in tears is significantly lower than in CSF, indicating a minimal presence of the protein in this biofluid. This low concentration might contribute to the reduced RT-QuIC performance observed when using tears as a sample.

### Determination of PrP levels using a SIMOA based homebrew assay

For the determination of total PrP, we have developed homebrew assay using Quanterix Homebrew starter and development kits. In general, manufacturer´s protocol was followed for the development of the kit. Briefly, among several antibody combinations for capture and detection, the SAF32 antibody-bead and 8G8 antibody-detection combination was selected. The standard curve exhibited a dynamic range extending up to 384 ng/mL, allowing for broad applicability across various sample types.

Following the development of the PrP assay, we assessed multiple dilution ratios for CSF and TF to determine the assay’s sensitivity threshold and to minimize possible matrix interferences. Based on this optimization, we selected a 1:120 dilution for CSF and a lower 1:50 dilution for TF. Prior to analysis, all samples were fully thawed and then centrifuged at 5,000×*g* for 5 min. Subsequently, they were allowed to equilibrate at room temperature for 30 min, in accordance with general procedural recommendations provided by Quanterix assay protocols.

### Statistical analysis

To compare multiple groups, parametric datasets were analyzed using one-way ANOVA followed by Tukey’s post hoc test, while non-parametric datasets were assessed using the Kruskal–Wallis test with Dunn’s multiple comparisons. For comparisons between two groups, the unpaired Student’s t-test was utilized for data with normal distribution, whereas the Wilcoxon Mann–Whitney test was applied for data that did not meet normality assumptions.

All statistical evaluations were conducted using GraphPad Prism version 10.0. Significance thresholds were set as follows: * p < 0.05, ** p < 0.01, *** p < 0.001, **** p < 0.0001.

## Results

### Modification and optimization of the RT-QuIC assay

To enhance the sensitivity of the CSF RT-QuIC assay for detecting amyloidogenic PrP conformers in TF [7, 8], we modified our protocol [29]. Considering that a point mutation in a full-length human recombinant (rec) PrP substrate might improve seeding conversion efficiency, we generated several FL Hu rec PrP substrates with point mutations at E200K (associated with genetic CJD) and D178N (associated with FFI). We then analyzed their application in the assay, comparing them to FL Hu and chimeric hamster-sheep rec PrP [7].

To ensure a reliable seeding reaction, we estimated the self-aggregation potential of different rec PrP substrates. We subjected CSF samples from controls (without prion disease), reactions without seed (without CSF), and without rec PrP substrate (n = 5 per condition) to RT-QuIC analysis. In contrast, to rec PrP D178N substrate, which showed self-aggregation as indicated by false-positive seeding reactions after 10-15 h in non-prion control samples or after 25 h without seed (no CSF sample added), the other substrates remained negative (Suppl. Fig. S1 A-D). Consequently, rec PrP D178N was excluded from further analysis. Generally, reaction without seed may undergo over a longer period of time (more than 80 h) spontaneously misfolding and aggregating independently from the kind of rec PrP substrate. When no sample (seed) is added, there are no endogenous proteins or other matrix components to stabilize the buffer environment, so the substrate may be more prone to non-specific nucleation. Therefore, the presence of sample material (e.g. control CSF or TF) is required in the RT-QuIC to stabilize the reaction (for example via protein binding, ionic strength, or surface shielding), preventing false positives.

Furthermore, we verified the stability of the assay with various substrates and the resistance to repeated CSF freezing and thawing. Five CSF samples from sCJD patients underwent multiple freezing and thawing cycles (up to 12 cycles). Using the same CSF samples at T0 without freezing and thawing as a reference value, we selected additional time points after four (T4), eight (T8), and twelve (T12) cycles of freezing and thawing. All reactions with chimeric hamster-sheep, FL Hu, and FL Hu E200K substrates remained positive without any significant changes in the seeding kinetics indicating their resistance to at least 12 cycles of freezing and thawing (Suppl. Fig.S2 A1-3-C1-3).

Subsequently, we assessed the seeding conversion efficiency of various rec PrP substrates in CSF samples. Signal responses from patients with sCJD, gCJD E200K, and FFI were quantified using semi-quantitative seeding parameters, including lag-phase and relative area under the curve (AUC). Notably, the FL Hu E200K substrate demonstrated the highest seeding conversion activity in sCJD, gCJD E200K, and FFI patients, as evidenced by the shortest lag-phase and the highest AUC values (Suppl. Fig. S3 A1-3-C1-3). Importantly, non-CJD control samples exhibited negative RT-QuIC responses across all substrates (Suppl. Fig. S3 D).

### CSF RT-QuIC diagnostic accuracy using different substrates

To assess the diagnostic accuracy of various rec PrP substrates, we tested CSF samples from patients with definite and probable CJD (including frequent and rare subtypes like MM/MV2C and VV1), as well as gCJD E200K, FFI, and GSS and non-prion disease control patients. Whereas all substrates exhibited a specificity of 100% (all controls remained negative), FL Hu E200K showed the highest diagnostic accuracy for CJD of 93% compared to hamster-sheep (78%) and FL Hu (77%) substrate (Table 1). In the context of atypical sCJD, the utilization of the FL Hu E200K substrate enhanced overall sensitivity substantially, escalating from 54 to 62% and in genetic prion diseases (gPD) specifically in a relatively large cohort of FFI patients, rising from 19 to 75% (Table 1).

**Table 1:** Diagnostic accuracies of different rec PrP substrates in the CSF RT-QuIC assay with 95% confidence interval (CI)

Group	Hamster-sheep	FL Hu	FL Hu E200K
Total CJD	101/129 (78%) [69.8–84.9%]	83/108 (77%) [67.6–84.4%]	120/129 (93%) [87.4–96.2%]
Definite/probable sCJD	86/111 (77%) [68.0–84.0%]	68/90 (76%) [65.6–84.3%]	102/111 (92%) [85.4–96.0%]
Genetic CJD E200K	15/18 (83%) [61.9–94.4%]	15/18 (83%) [61.9–94.4%]	18/18 (100%) [84.6–100.0%]
MM/MV1 (+ 2C)	36/43 (84%) [70.9–91.8%]	33/43 (77%) [62.2–87.5%]	42/43 (98%) [88.0–99.9%]
VV2	6/8 (75%) [40.9–92.9%]	7/8 (87%) [52.9–97.8%]	8/8 (100%) [67.6–100.0%]
MV2K	3/3 (100%) [43.9–100.0%]	3/3 (100%) [43.9–100.0%]	3/3 (100%) [43.9–100.0%]
VV1	3/8 (38%) [13.9–68.4%]	–	7/8 (88%) [53.6–98.3%]
MM/MV2C	7/13 (54%) [28.0–78.7%]	–	8/13 (62%) [35.5–82.3%]
FFI	6/32 (19%) [9.2–35.6%]	8/22 (36%) [18.0–59.2%]	24/32 (75%) [57.6–87.6%]
GSS	1/3 (33%) [6.1–79.2%]	1/2 (50%) [9.5–90.5%]	3/3 (100%) [43.9–100.0%]
Non-prion diseases	0/62 (100%) [94.2–100%]	0/62 (100%) [94.2–100%]	0/62 (100%) [94.2–100%]

Moreover, we evaluated the performance of two different rec FL Hu E200K batches by comparing their functional activity across all measured assays to determine whether batch-specific variability influenced the results. The analysis of 12 CSF samples (6 positives and 6 negatives) revealed no significant differences between the two FL Hu E200K batches (Suppl. Fig. S6A, B).

### Detection of amyloidogenic PrP conformers in TF: definition of (pre)-analytical conditions

**Fig. 1:**
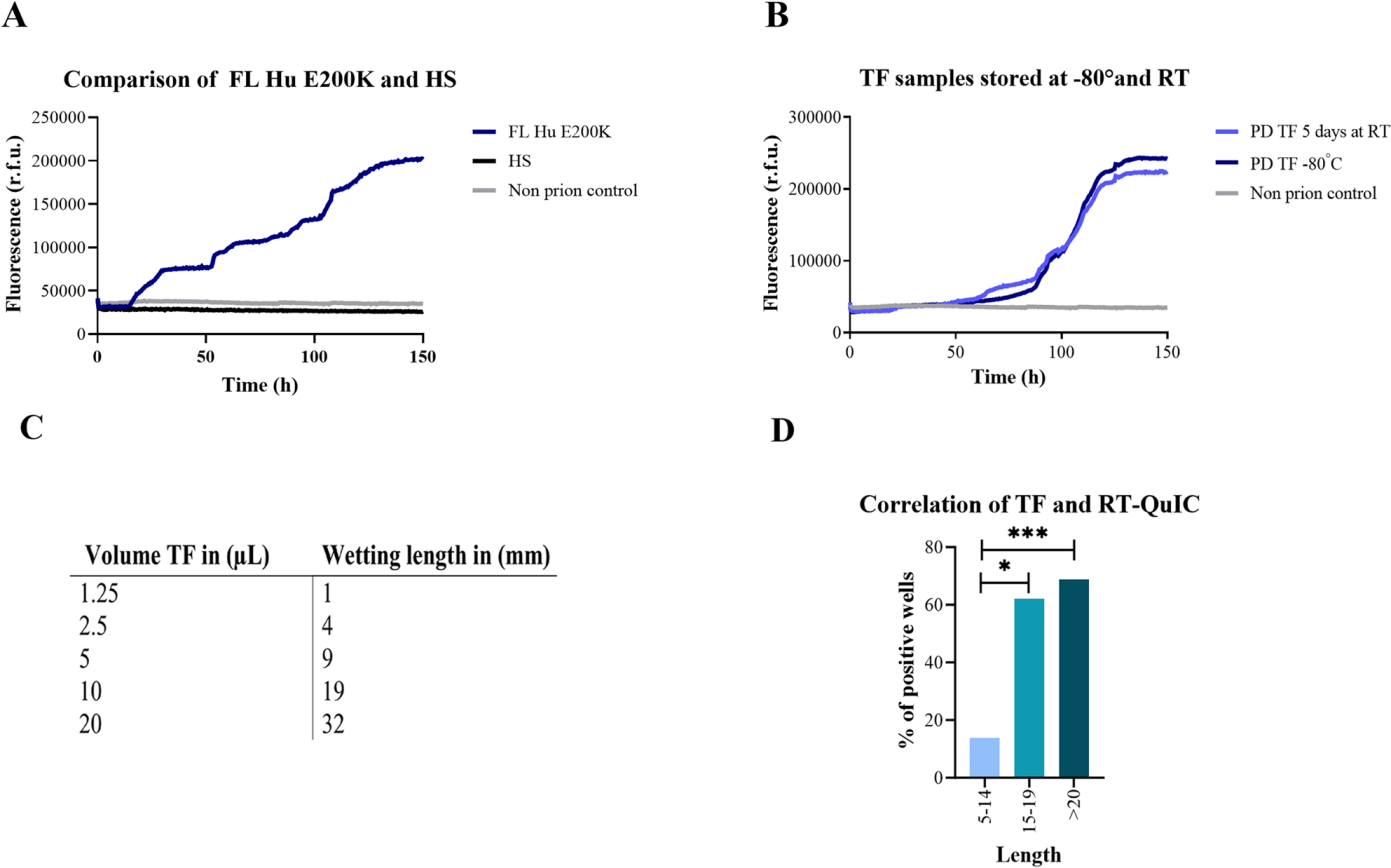
Seeding conversion kinetics, stability and (pre)-analytical conditions of TF RT-QuIC. **A** Comparative RT-QuIC kinetic curves of sCJD TF samples (n = 4) using HS substrate (black line) and FL Hu E200K substrate (blue line). The HS substrate showed no measurable seeding activity over 150 h. **B** Stability test of sCJD TF samples (n = 4) using the RT-QuIC assay with FL Hu E200K substrate after storage at -80 °C (dark blue line) and room temperature for 5 days (light blue line). Both storage conditions yielded comparable RT-QuIC signal responses, confirming the assay’s robustness. Non-prion controls remained negative independent from the kind of substrate and the storage time. **C** Correlation of Schirmer Tear Test strip wetting length with TF volume (µL). **D** Proportion of positive RT-QuIC wells as a function of wetting length from the Schirmer strips (n = 20). A minimum wetting length of 15–25 mm is recommended for reliable diagnostic outcomes. Statistical analysis was done by one-way ANOVA followed by Tukey's multiple comparison test (* p-value < 0.05, *** p-value < 0.001)

Based on its promising sensitivity in the CSF and the aforementioned signal response characteristics, we selected the FL Hu E200K substrate for the TF RT-QuIC assay. We compared the seeding response curves of TF sCJD-seeded reactions using this substrate with those from the chimeric hamster-sheep substrate. In contrast to full-length human E200K substrate, the chimeric hamster-sheep substrate showed no seeding response after 150 h of measurement (Fig. 1A).

To assess the stability of prion seeding in TF samples at room temperature (RT), we collected TF samples from four patients with sCJD and stored aliquots at -80 °C and at RT for five days until the analysis. No significant differences were observed in the signal kinetic curves or in other RT-QuIC parameters of interest, including lag-phase and AUC (data not shown), suggesting that prion activity remains stable for at least up to 5 days at RT (Fig. 1B).

A consistent positive signal response in the RT-QuIC was observed with FL Hu E200K rec PrP substrate when more than 15 mm liquid-length on the strip were applied. This length corresponds to approximately 10–20 µL of TF (Fig. 1C, D). Suggestions to increase the reproducibility of the TF-RT-QuIC are listed in Table S2 based on this information (Table S2).

### Detection of total PrP levels in CSF and TF

**Fig. 2:**
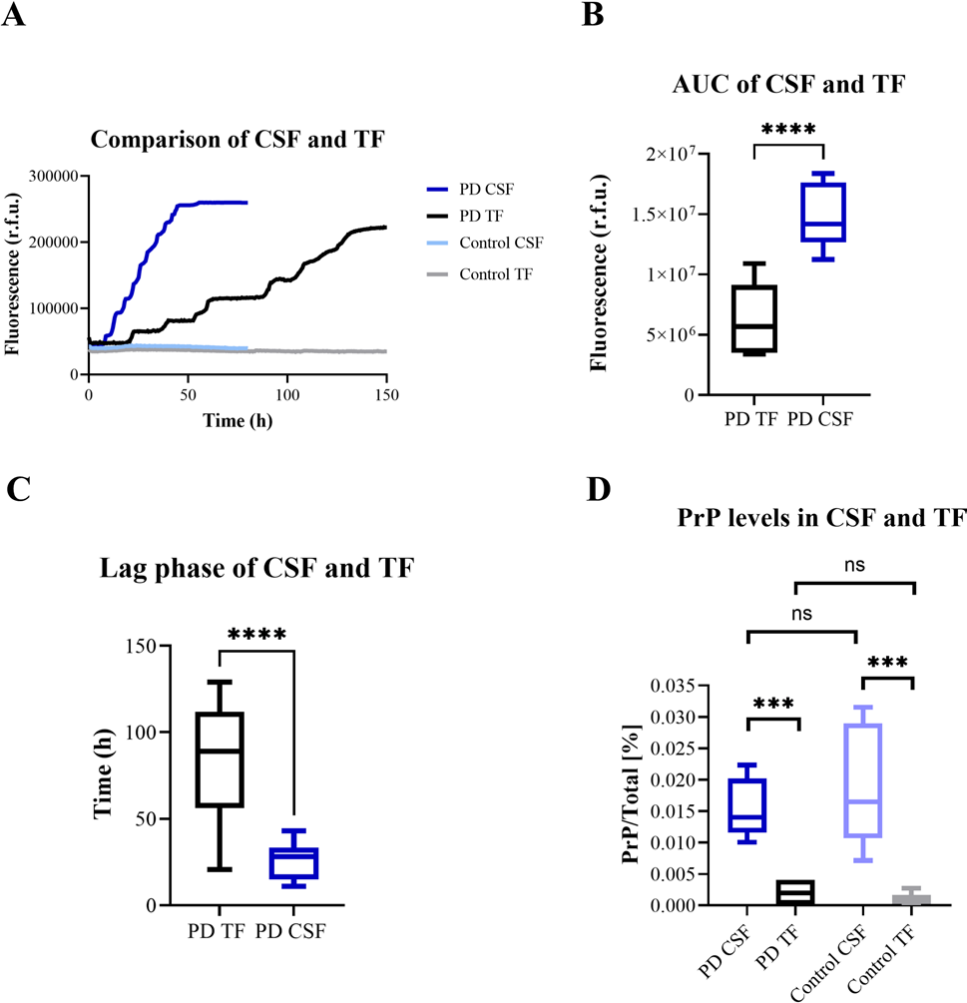
Comparison of RT-QuIC performance and PrP levels in CSF and TF samples of the same individual using FL Hu E200K as a substrate. **A** Fluorescence intensity curves from RT-QuIC analysis comparing prion disease (PD) CSF (n = 1 GSS, n = 1 T183A, n = 5 sCJD) (light blue) and TF (n = 1 GSS, n = 1 T183A, n = 5 sCJD patients) (dark blue); n = 7 per group. Non-prion controls remained negative independent from the kind of bodyfluid (TF or CSF). **B** Area under the curve (AUC) analysis reveals significantly higher cumulative fluorescence in CSF compared to TF (Student’s t-test ****p < 0.0001). **C** Lag-phase duration comparison demonstrates significantly shorter lag phases in CSF compared to TF (****p < 0.0001). **D** Total PrP level, measured as a percentage of total protein using SIMOA technology, were significantly higher in CSF than TF for both PD patients and controls (n = 4) (Student’s t-test ***p < 0.001

The comparative kinetic RT-QuIC curves from CSF and TF from sCJD (same individuals) are displayed in Fig. 2A. The AUC was significantly lower and the lag-phase was significantly longer in TF samples compared to those seeded with CSF (Fig. 2B, C).

To compare the total PrP level in TF and CSF, we employed a homebrew assay for PrP based on SIMOA technology and measured total PrP in paired TF and CSF samples from prion disease patients and non-prion disease controls. Since TF-proteins were eluted into RT-QuIC buffer (diluting the sample), we indicated the amount of total PrP as a percentage of total protein.

Interestingly, we observed a significantly higher percentage of total PrP in the CSF than in TF but no significant difference between prion diseases and non-prion disease groups (Fig. 2D).

### Impact of disease stage on the seeding conversion activity in TF RT-QuIC

**Fig. 3:**
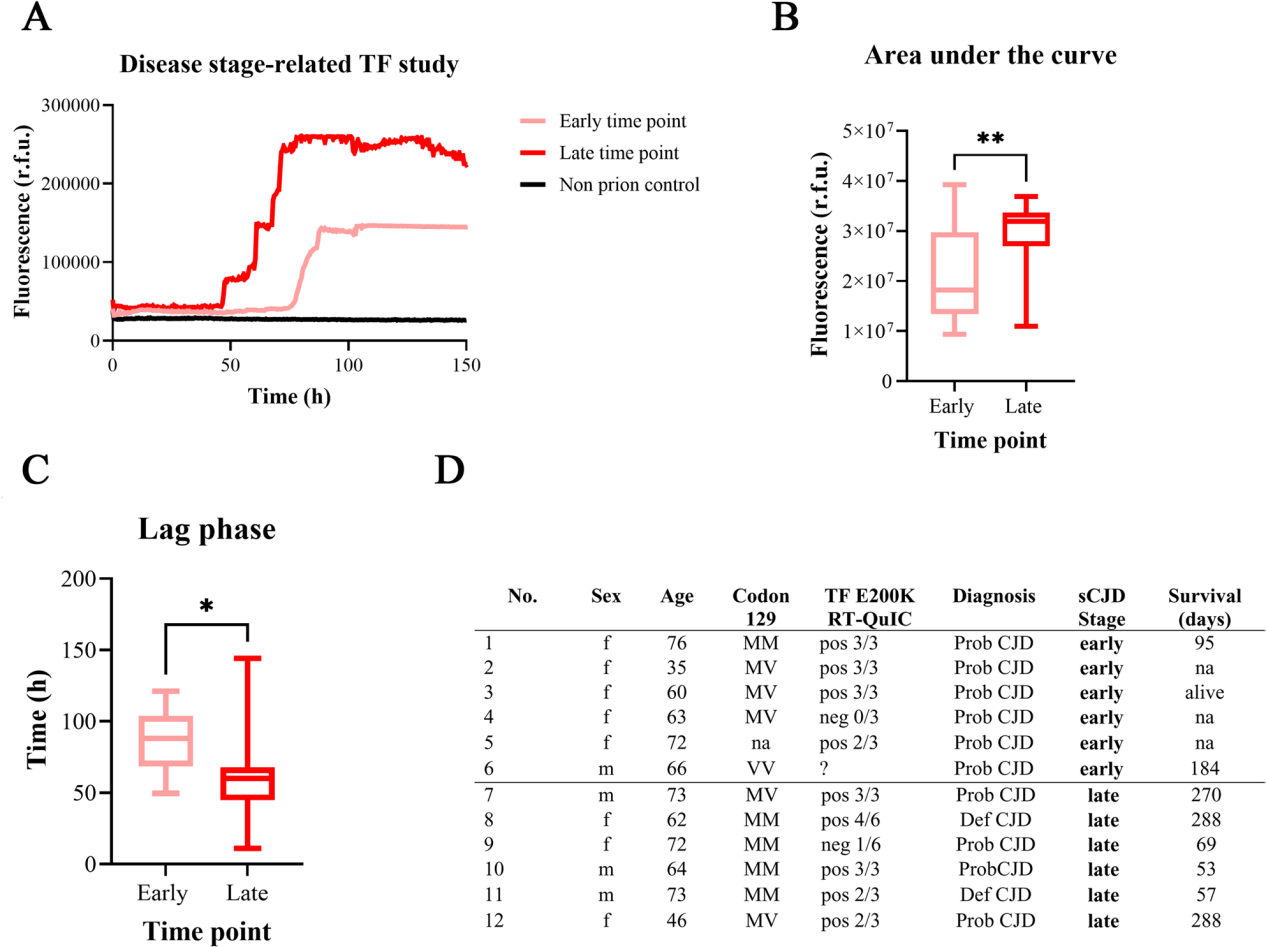
Influence of early- and late-stage time points on RT-QuIC responses in TF. **A** Fluorescence intensity curves from the RT-QuIC assay comparing early (light red) and late (dark red) time points in TF samples (n = 6, sCJD per group) from different individuals. The late-stage time point exhibits higher overall fluorescence intensity, indicating increased seeding activity. Non-prion disease controls remained negative independent of the disease stage. **B** Area under the curve (AUC) analyses comparing early and late-stage time points. A significant increase (Student’s t-test, p < 0.01) in AUC at the late-stage time point reflects enhanced cumulative seeding activity. **C** Lag phase duration for early and late-stage time points. The late-stage time point demonstrates a significantly shorter lag phase (Student’s t-test, p < 0.05), indicating faster amplification kinetics of amyloidogenic PrP conformers in the RT-QuIC assay. **D** Demographic characteristics of both groups

RT-QuIC signal responses were compared based on the timing of TF sampling (either at early or late disease stages) and RT-QuIC kinetic curves were illustrated as mean values (Fig. 3A). To quantify RT-QuIC signal responses, AUC values were computed, indicating that a later disease stage is associated with a more pronounced signal response (Fig. 3B). TF collected at a later disease stage exhibited a significantly shorter lag phase compared to those collected at disease onset (Fig. 3C). Demographic characteristics of both groups are shown (Fig. 3D).

### Effect of gender, age and codon 129 MV genotype

We investigated the impact of age, gender and *PRNP* codon 129 MV genotype on the signal response in the TF RT-QuIC assay. No significant differences were observed in the seeding parameters of interest, including lag phase and AUC, between the female and male groups (Supplementary Figure S4 A1-3), males or females aged under 50 years old and those aged 50 years and above (Supplementary Figure S4 B1-3, C1-3). No effect of the *PRNP* codon 129 MV genotype was seen (Suppl. Fig. S5A-C).

### Validation of diagnostic accuracy of TF RT-QuIC and the determination of the overall diagnostic accuracy

To validate the diagnostic accuracy of the TF RT-QuIC from our previous study (cohort 1) [30] in a new independent cohort 2, we collected additional, well-characterized TF samples and analyzed them under the same experimental conditions (rec PrP substrate and protocol).

First, we assessed the overall diagnostic accuracy of FL Hu E200K rec PrP substrate in TF samples and combined two different cohorts to achieve a statistically powerful sample size.

Overall, the study involved patients with sCJD, patients with genetic prion diseases, and non-prion disease controls. FL Hu E200K rec PrP substrate exhibited an overall sensitivity of 85% in sCJD and 64% in genetic prion diseases (Table 2). From 184 non-prion controls, one sample was tested positive in two out of three RT-QuIC reactions resulting in a specificity of 99.5% (Table 2) (Suppl. Fig. S7). The **c**linical assessment supports a diagnosis of mild to moderate Alzheimer’s dementia, with an MMSE score of 21/30 at both baseline and one-year follow-up. CSF analysis shows a typical Alzheimer’s disease biomarker profile with reduced Aβ1-42 (593 pg/ml), an abnormal Aβ ratio (Aβ1-42/1–40 ratio × 10) of 0.49, and elevated phospho-Tau (85.0 pg/ml), while total Tau remains within normal range (386 pg/ml), corresponding to an A + /T + /N − profile. EEG reveals mild general slowing without periodic sharp wave complexes. MRI demonstrates mild global atrophy and mild cerebral small vessel disease (Fazekas grade I), with no signs of restricted diffusion.

**Table 2:** Diagnostic accuracies of TF RT-QuIC using the FL Hu E200K rec PrP substrate in sporadic and genetic prion diseases with 95% confidence interval (CI)

Group	1st Cohort*^1^ sensitivity /specificity + [95%CI]	2nd Cohort sensitivity /specificity + [95%CI]	Total sensitivity /specificity + [95%CI]
sCJD (symptomatic)	13/15 (87%) [63.0–97.1%]	16/19 (84%) [61.1–95.5%]	29/34 (85%) [69.9–93.6%]
Genetic prion diseases (symptomatic)	**4/5 (80%) [37.6–96.4%]**	**3/6 (50%)** [18.0–81.0%]	**7/11 (64%)** [35.3–91.9%]
FFI	2/2 (100%) [34.2–100%]	1/3 (33%) [7.5–70.1%]	3/5 (60%) [23.1–88.2%]
GSS	1/1 (100%) [20.7–100%]	2/3 (67%) [9.4–99.2%]	3/4 (75%) [30.1–95.4%]
T183A	1/2 (50%) [9.5–90.5%]	–	1/2 (50%) [9.5–90.5%]
HMC^*2^	**6/8 (75%)** [40.9–92.9%]	**10/20 (50%)**[29.9–70.1%]	**16/28 (57%)**[39.0–73.6%]
FFI	2/2 (100%) [39.8–100%]	5/8 (62.5%) [32.6–85.8%]	7/10 (70%) [41.7–98.3%]
5-OPRI	2/2 (100%) [39.8–100%]	2/2 (100%) [39.8—100]	4/4 (100%) [51.0—100]
GSS	2/3 (67%) [25.1–90.0%]	2/2 (100%) [39.8—100]	4/5 (80%) [37.6–96.4]
E200K	0/1 (0%) [0.0–97.5%]	0/7 (0%) [0.0—41.0]	0/8 (0%) [0.0–35.4]
G114V	–	1/1 (100%) [20.7–100%]	1/1 (100%) [20.7%–100]
Non-prion diseases*^3^	**0/68 (100%) [94.7–100%]**	**1/118 (99.2%) [95.5–99.9%]**	**1/184 (99.5%) [96.9–99.9%]**

^*^^1^Cohort 1 previously reported (Schmitz et al., 2023)

^*^^2^HMC = healthy mutation carriers (non-symptomatic persons at risk)

^*^^3^Diagnosis: Healthy individual, immune-mediated disorders, AD, MS, ischemia, Parkinson disease, mixed dementia, VD and others

Secondly, we validated the diagnostic accuracy obtained in our previous cohort [30] by analyzing independently collected TF samples via RT-QuIC (cohort 2).

Interestingly, we could confirm the diagnostic accuracy in cohort 2. Patients with prion diseases revealed in 16/19 (84%) a positive TF RT-QuIC response reactions for sCJD which is comparable to cohort 1 (13/15 (87%) (Table 2). 99.2% of all controls from cohort 2 remained negative confirming a very high diagnostic specificity of the TF RT-QuIC (Table 2).

Finally, we compared the detection of TF-RT-QuIC signals in HMC (Table 2). In the first cohort, the test was positive for TF samples from 6 out of 8 asymptomatic PRNP mutation carriers (75%). In the second cohort, signal detection in the TF of HMCs was confirmed in 10 out of 20 patients (50%), yielding an overall accuracy of 57% for the TF RT-QuIC test in HMCs (Table 2, Table S2).

## Discussion

Seeded aggregation assays, such as RT-QuIC, have been suggested as a class of biomarker assays for diagnosis of prion diseases. Minuscule amounts of amyloidogenic PrP conformers can be amplified in specimens to a detectable level, e.g., in CSF and other tissues. These methods entered the field of clinical diagnostic assays in prion diseases [11, 35], and are in the development stage for other neurodegenerative disorders such as synucleinopathies [5, 13, 28].

For the development of a solid and reproducible test, there are several questions to be addressed, such as a robust substrate, optimal running conditions for test sensitivity and specificity and the best biomatrix to be studied. We hypothesized that rec PrP substrates containing prion disease associated mutations may facilitate the seeding conversion process of rec PrP substrate in the RT-QuIC assay. To test this hypothesis, we synthesized various rec PrP substrates with specific mutations. The rec PrP substrate carrying the E200K mutation displayed the best performance in the RT-QuIC with respect to test stability, consistency and diagnostic accuracy in CSF in prion diseases. Compared to previous studies applying different rec PrP substrates such as chimeric hamster-sheep, we could increase the diagnostic sensitivity of the CSF RT-QuIC to 93% for CJD patients and for FFI to 75% by utilizing the FL Hu E200K rec PrP substrate [7, 8, 31].

In particular, for the diagnostic of FFI cases the FL Hu E200K rec PrP substrate offers significant advantages compared to the chimeric hamster-sheep rec PrP [7, 31].

In terms of the broader diagnostic application for prion diseases, the utilization of FL Hu E200K substrate in CSF RT-QuIC demonstrated a sensitivity (93%) and specificity (100%) which is comparable to recent findings on the second generation of RT-QuIC (“Improved QuIC” or “IQ”). The IQ employs truncated hamster rec PrP (residues 90–231) showing a diagnostic sensitivity for CJD of 92–97%, a specificity of 100% and a very high reproducibility [10, 24, 25]. In addition, our sCJD cohort included a relatively high proportion of rare and slowly progressive sCJD subtypes (MM/MV2C and VV1, n = 21), showing a sensitivity of 62% and 88%, respectively. The sensitivity of most common MM/MV1, VV2, and MV2K subtypes was between 98 and 100%. In contrast, gCJD E200K and GSS analysis revealed a sensitivity of 100% in (n = 18 E200K and n = 3 GSS).

### Modification of RT-QuIC assay for the detection of prion seeding activity in TF

Various matrices have been discussed for biomarker assessment in CJD. CSF with its ability to reflect central nervous processes more accurately than peripheral fluids such as blood components [10] is most often used for biomarker research. Invasiveness and the potential risk due to lumbar puncture present a drawback of this method, especially concerning the need for longitudinal sample collection, as needed for research on reliable disease progression markers in clinical trials. PrP amplification assays have already been developed for detection of amyloidogenic PrP conformers in blood [6] and urine [15].

Blood-based assays have already been emerged as valuable tools in the diagnosis of variant Creutzfeldt–Jakob Disease*.* In addition to confirming clinical diagnoses [4], these tests have demonstrated the presence of prionemia during the asymptomatic (preclinical) phase of disease in humans [3].

The development of a sensitive and specific blood test capable of detecting misfolded prion protein (PrP^Sc^) during the incubation phase of sCJD would be particularly important for identifying asymptomatic carriers and reducing the risk of iatrogenic transmission through medical procedures and contaminated instruments.

In addition, new approaches using minimal-invasively accessible biomaterial, such as skin punch biopsy or olfactory mucosa have shown great potential [2, 17, 23, 37]. In search for a non-invasively accessible biofluid, we decided to evaluate the potential applicability of the methodology for TF for various reasons: similar to CSF, TF is low in protein content, in contrast to serum or plasma and might therefore have less pre-analytical problems compared to blood or tissue biopsies. Considering pre-analytical factors, it is also free of relevant blood contamination. Tear fluid can be safely collected using the Schirmer method, even by non-professionals. Proteins are eluted from the strips by buffer treatment and centrifugation to collect the eluate (Suppl. Figure S8).

Regarding the potential infectivity of RT-QuIC end-products from TF or CSF seeded reactions, available evidence indicates that the amplified fibrils are poorly infectious. Raymond et al. [27] showed that Tg66 mice inoculated with PK-resistant RT-QuIC products generated from sCJD-CSF (post-mortem) or brain material (amplified with hamster substrate) did not develop clinical disease and lacked detectable PrP^Sc^. Ongoing studies using TF and CSF from CJD patients—including samples seeded with wild-type and E200K mutant PRNP—are still underway, so the infectivity of these RT-QuIC end-products cannot yet be fully assessed.

Occurrence of relevant biomarkers in TF for neurodegeneration like neurofilament light chain or alpha-synuclein in TF has been recently reported in Parkinson’s disease [14, 16].

In our study, we detected seeding activity in TF in sCJD and in genetic prion diseases using our modified CSF protocol. When utilizing sufficient sample volumes, approximately 15µL, we observed that the TF RT-QuIC demonstrates remarkable stability and robustness. This was evident when the TF sample was incubated at RT for up to 5 days, comparable to the characteristics of CSF RT-QuIC [7].

In addition, we could show that the TF RT-QuIC is very reproducible among different cohorts. We confirmed the diagnostic accuracy of a previously published cohort [30] in a second cohort under the same experimental conditions. The overall diagnostic sensitivity of the TF RT-QuIC for sCJD is 85%, which is similar to the sensitivity of the CSF RT-QuIC using hamster-sheep substrate [7, 8] but lower compared to the CSF RT-QuIC with FL E200K substrate.

In our HMC cohort 1, the test showed a high positivity rate of 75%, whereas the second cohort demonstrated a notably lower detection rate of 50%. This discrepancy may be attributed to differences in sample size, prion disease subtypes, and other demographic factors such as age or the time to clinical onset. Other studies have reported that the prodromal window—from CSF RT-QuIC positivity to disease onset—was approximately 1 year in an E200K individual homozygous (V/V) at PRNP codon 129, and 2.5 to 3.1 years in two codon 129 heterozygotes (M/V) [33]. An other study, detected seeding activity in CSF in four presymptomatic CSF samples from three E200K carriers; one converted within 2 months while two remain asymptomatic after at least 3 years' follow-up [22]. Longitudinal studies in HMC are essential to assess whether TF positivity serves as an early biomarker which can roughly predict the disease onset.

A positive test result was observed in TF from 1 out of 184 control subjects, diagnosed as AD showing a pregnant amyloid and tau pathology. However, mutation in *PRNP* could not be excluded. A false positive RT-QuIC result in an AD patient may arise due to non-specific aggregation of rec PrP or cross-seeding by other misfolded proteins present in AD pathology. This highlights the assay’s potential limitations in specificity across neurodegenerative disorders.

The diagnostic specificity of 99.5% is in agreement with most CSF RT-QuIC studies (specificity of 99–100%) [1, 7, 19]. Similar to other CSF studies, we did not observe an impact of age, gender or codon 129 genotype [8] highlighting the diagnostic potential of the TF RT-QuIC when considering the recommendations (Table S2). However, the stage of the disease may impact the RT-QuIC signal response, which is in concordance with previous CSF studies [7].

Differences in RT-QuIC performance between CSF and TF may stem from the distinct biochemical composition of each matrix. We interpreted the lower AUC and extended lag-phase in TF RT-QuIC with a lower amount of total PrP in TF compared to CSF. Additionally, TF and CSF exhibit distinct biochemical profiles, which can also have important implications in signal kinetic. TF contains significantly higher total protein concentrations (6–11 mg/mL) than CSF (< 0.5 mg/mL), largely due to a few high-abundance proteins such as lysozyme, lactoferrin, lipocalin, and secretory IgA (CSF proteins are more diluted and primarily include albumin, immunoglobulins) [39]. These dominant proteins may interfere with protein-based assays through non-specific binding or surface interactions.

Ionic composition also differs between the fluids. TF has higher native osmolarity (~ 300 mOsm) and distinct ion levels (e.g., high K^+^ and Cl^−^), which can influence protein folding and aggregation kinetics [34]. Furthermore, TF contains various mucins and glycoproteins (largely absent in CSF), which can sterically hinder aggregation or bind proteins non-specifically [36].

In conclusion, we report here a modified RT-QuIC assay for detection of PrP seeding activity in CSF and TF in patients with sporadic and genetic CJD. Overall, the seeding activity and diagnostic accuracy of the RT-QuIC in TF were lower compared to CSF, which is a limitation of the TF RT-QuIC. TF, as a matrix, offers various advantages over CSF. Due to its non-invasiveness TF improves patient comfort and safety, especially in fragile or elderly individuals. The collection process poses minimal infection risk to healthcare personnel and most importantly it can be done repeatedly to monitor disease progression or response to interventions. Additionally, TF RT-QuIC also facilitates early diagnosis in atypical or rapidly progressive dementias, where rapid confirmation of prion disease can guide clinical decisions, infection control measures, and family counseling. Thus, TF RT-QuIC represents to our opinion a very valuable, non-invasive diagnostic tool that can extend CSF RT-QuIC diagnostic.

## Supplementary Information

Below is the link to the electronic supplementary material.

Supplementary Figure 1: Validation of the self-aggregation characteristics of different rec PrP substrates. Five reactions without CSF seed (red line), five without substrate (blue line), and five with non-prion CSF controls (green line) using chimeric hamster–sheep (A), FL Hu (B), and FL Hu E200K (C) substrates showed a negative seeding response (flat lines) after 80 h of measurement. RT-QuIC reactions using the rec PrP FL Hu D178N (D) substrate, either seeded with control CSF samples from non-prion disease cases (green line) or without CSF seed (red line), indicated self-aggregation properties of this substrate.

Supplementary Figure 2: RT-QuIC reactions with chimeric hamster-sheep, FL Hu, and FL Hu E200K substrates across various freezing and thawing cycles. Mean fluorescence values of RT-QuIC assay with chimeric hamster-sheep (A1), FL Hu (B1), and FL Hu E200K (C1) substrates seeded with sCJD CSF (n = 5) after different cycles of freezing and thawing (T = 0, 4, 8 and 12). Neither the chimeric hamster-sheep substrate (A), the FL Hu (B), nor the FL Hu E200K (C) substrate reactions revealed significantly different signal responses after 12 freezing and thawing cycles in the area under the curve (AUC) (A2, B2 and C2) and lag-phase (A3, B3 and C3)

Supplementary Figure 3: RT-QuIC assays seeded with CSF samples from sCJD, gCJD E200K, and FFI patients were used to compare the seeding conversion efficiencies of hamster–sheep (HS), FL Hu, and FL Hu E200K rec PrP substrates. Positive signal response curves of sCJD samples (n = 43) (A1), gCJD E200K samples (n = 16) (B1), and FFI patient samples (n = 22) (C1) are shown for each rec PrP substrate (chimeric HS, FL Hu, and FL Hu E200K). The area under the curve (AUC) (A2, B2, C2) and the lag phase (A3, B3, C3) were used for quantification. For sCJD and gCJD, the rec PrP FL Hu E200K substrate showed the highest seeding conversion efficiency. No seeding conversion was observed in CSF samples from control subjects (n = 44) (D).

Supplementary Figure 4: Impact of age and gender on the TF RT-QuIC signal response. The kinetic curves of positive TF RT-QuIC reactions from male and female individuals with prion diseases (PD) were compared across different age groups. Mean fluorescence values from TF RT-QuIC assays were analyzed in female (n = 11; sCJD = 7, FFI = 2, GSS = 1, fCJD [T183A] = 1) and male (n = 11; sCJD = 9, GSS = 1, FFI = 1) prion disease samples (A1). Females were further categorized into younger (<50 years, n = 4; sCJD = 3, fCJD [T183A] = 1) and older (>50 years, n = 6; sCJD = 6) groups (B1). Males were similarly grouped into younger (39 years, GSS = 1) and older (53–70 years, sCJD = 7, GSS = 1, FFI = 1) groups (C1). The analysis focused on lag phase duration (2) and area under the curve (AUC) values (3). Neither sex (A1–3) nor age group (B1–3, C1–3) showed significant differences in TF RT-QuIC signal responses.

Supplementary Figure 5: Impact of the *PRNP* codon 129 MV genotype in sCJD on the TF RT-QuIC signal response. A) Comparison of kinetic curves of positive TF RT-QuIC reactions of different *PRNP* codon 129 MV genotypes in sCJD (MM, MV, VV). B-C) Applying quantitative parameters, such as the area under the curve (AUC) and duration of the lag-phase we observed no significant differences in signal response in the TF RT-QuIC assay among different *PRNP* codon 129 MV genotypes (n = 4 per group)

Supplementary Figure 6: RT-QuIC seeding kinetics showing batch-to-batch comparison of recombinant FL Hu E200K substrate. A) Signal responses, seeded with CSF from 6 CJD cases (each 3/3 positive) and 6 non-prion controls (each 0/3 negative), using FL Hu E200K substrate from both batch 1 and batch 2, reveal almost identical kinetic curves. B) Calculation of the area under the curve (AUC) values of sCJD seeded reactions indicated no significant differences between the two batches

Supplementary Figure 7: RT-QuIC seeding kinetics of a false positive AD-patient. The RT-QuIC was seeded with TF from a non-prion control. Two out of three reactions were considered as positive. The clinical assessment supports a diagnosis of mild to moderate Alzheimer’s dementia

Supplementary Figure 8: Procedure of tear fluid collection and strip extraction. A 5 × 35 mm strip was placed on the outer corner of the lower eyelid for 8–10 min, following the standard Schirmer test for tear production. Samples were frozen within 30 min and stored at − 80 °C without further treatment for storage. For protein isolation, samples were eluted by cutting at least 15 mm of the strip into 5 mm pieces, placing them in a 1.5 mL tube with 100 μL RT-QuIC buffer, vortexing for 1 min, and incubating for 30 min. Strips were then transferred to perforated 0.5 mL tubes placed above the 1.5 mL tubes and centrifuged at 14,000 rpm (Eppendorf centrifuge 5810 R), 4 °C for 30 min to collect the extract. Samples from both eyes were pooled for analysis.

Supplementary Material 9

Supplementary Material 10

Supplementary Material 11

## Data Availability

The datasets generated and/or analyzed during the current study are available from the corresponding author upon reasonable request.

## Abbreviations

CSF: Cerebrospinal fluid
FFI: Fatal familial insomnia
FL Hu: Full-length human
HMC: Healthy mutation carriers
PrP: Prion protein
PrPC: Cellular PrP
PrP^Sc^: PrP scrapie
RT-QuIC: Real-time quaking-induced conversion
rec PrP: Recombinant PrP
sCJD: Sporadic Creutzfeldt–Jakob disease
TF: Tear fluid
