# Supplementary figures and images for: Advancing prion diagnostics: full-length human E200K RT-QuIC substrate facilitates prion detection in tear fluid and improves sensitivity in cerebrospinal fluid
Source: Acta Neuropathol Commun. 2026 Jan 22;14:28. doi: 10.1186/s40478-025-02212-8 (PMC12849081; doi:10.1186/s40478-025-02212-8)

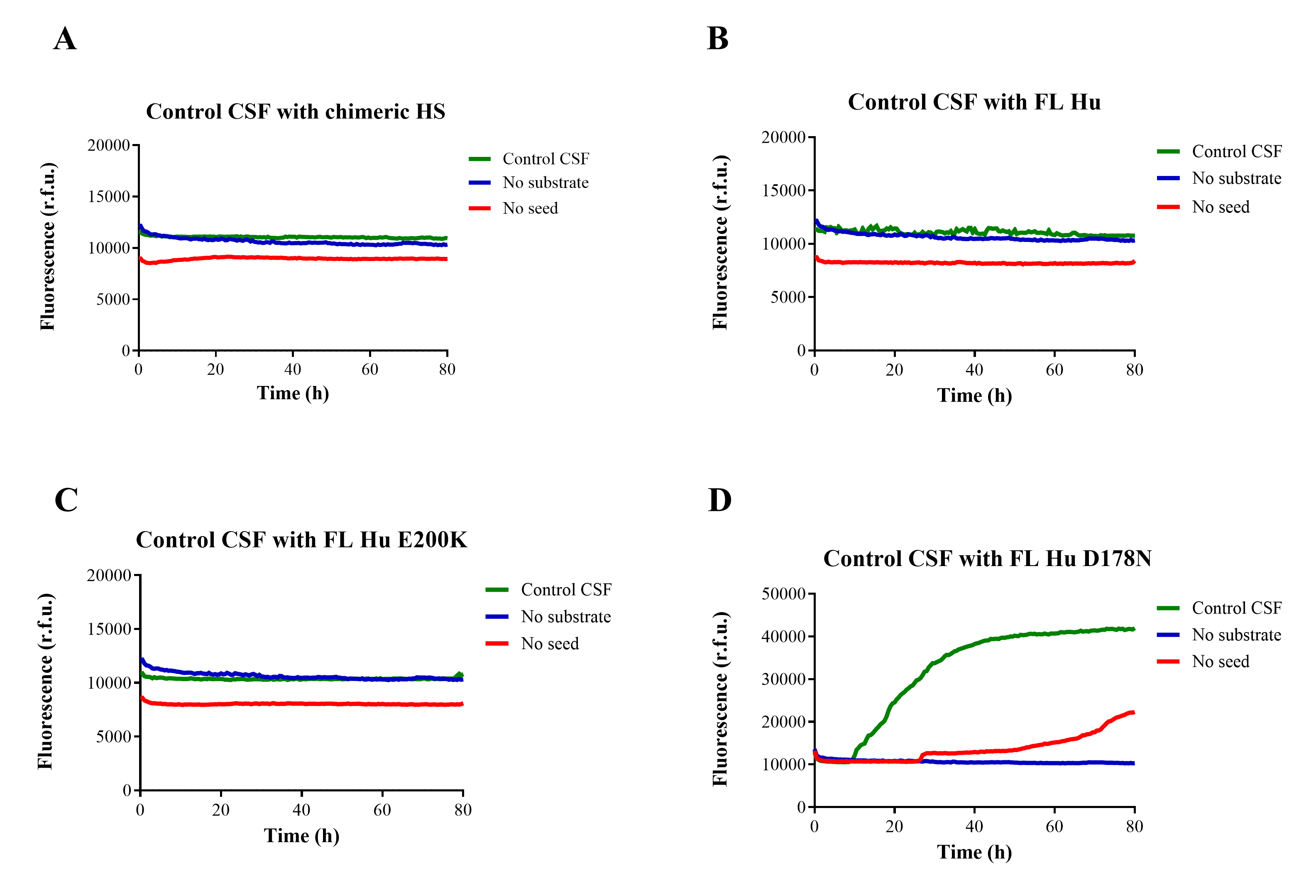

Supplement: Supplementary file 1 — Supplementary Figure 1: Validation of the self-aggregation characteristics of different rec PrP substrates. Five reactions without CSF seed (red line), five without substrate (blue line), and five with non-prion CSF controls (green line) using chimeric hamster–sheep (A), FL Hu (B), and FL Hu E200K (C) substrates showed a negative seeding response (flat lines) after 80 h of measurement. RT-QuIC reactions using the rec PrP FL Hu D178N (D) substrate, either seeded with control CSF samples from non-prion disease cases (green line) or without CSF seed (red line), indicated self-aggregation properties of this substrate. [file 40478_2025_2212_MOESM1_ESM.tif]

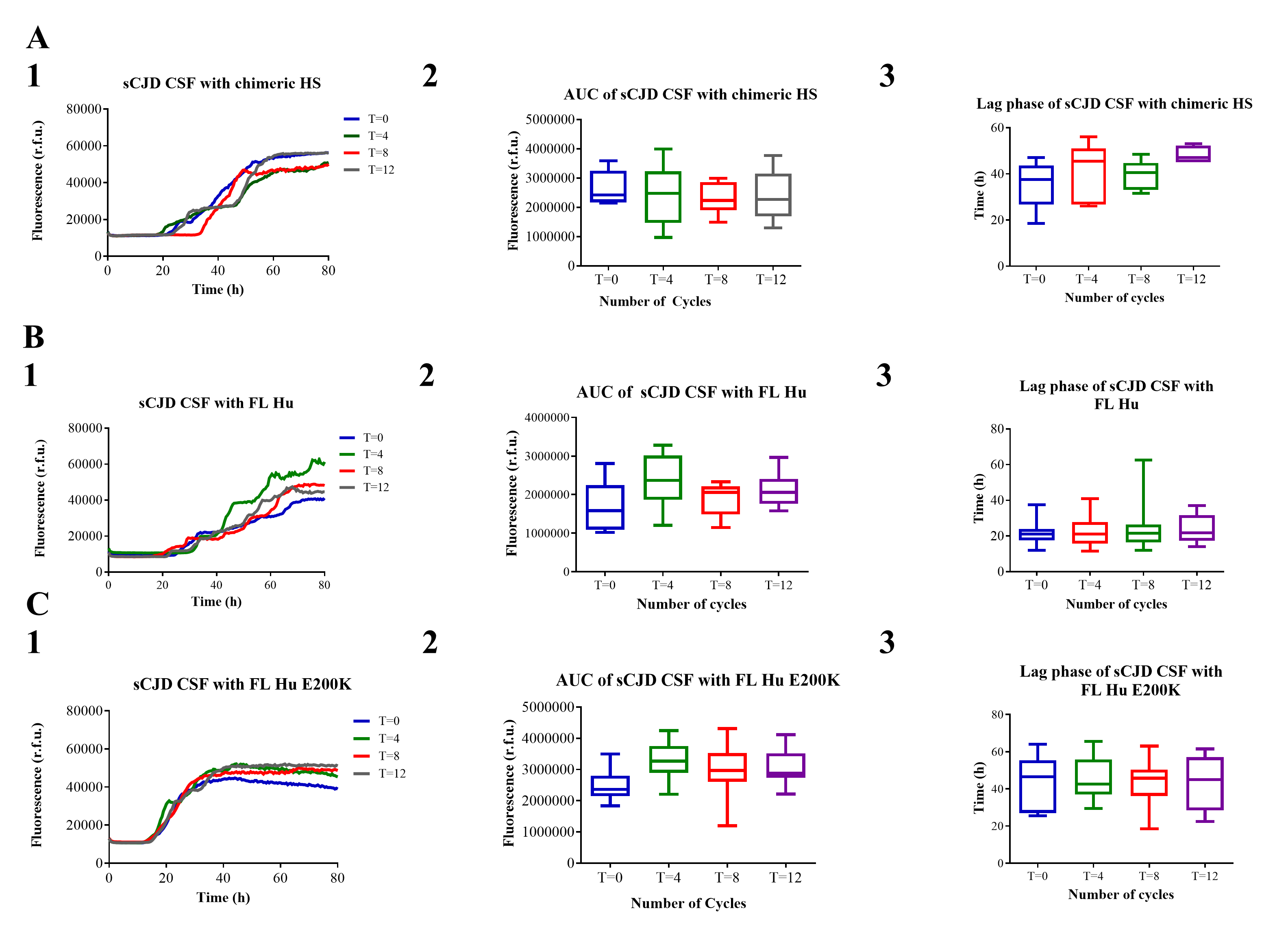

Supplement: Supplementary file 2 — Supplementary Figure 2: RT-QuIC reactions with chimeric hamster-sheep, FL Hu, and FL Hu E200K substrates across various freezing and thawing cycles. Mean fluorescence values of RT-QuIC assay with chimeric hamster-sheep (A1), FL Hu (B1), and FL Hu E200K (C1) substrates seeded with sCJD CSF (n = 5) after different cycles of freezing and thawing (T = 0, 4, 8 and 12). Neither the chimeric hamster-sheep substrate (A), the FL Hu (B), nor the FL Hu E200K (C) substrate reactions revealed significantly different signal responses after 12 freezing and thawing cycles in the area under the curve (AUC) (A2, B2 and C2) and lag-phase (A3, B3 and C3) [file 40478_2025_2212_MOESM2_ESM.tif]

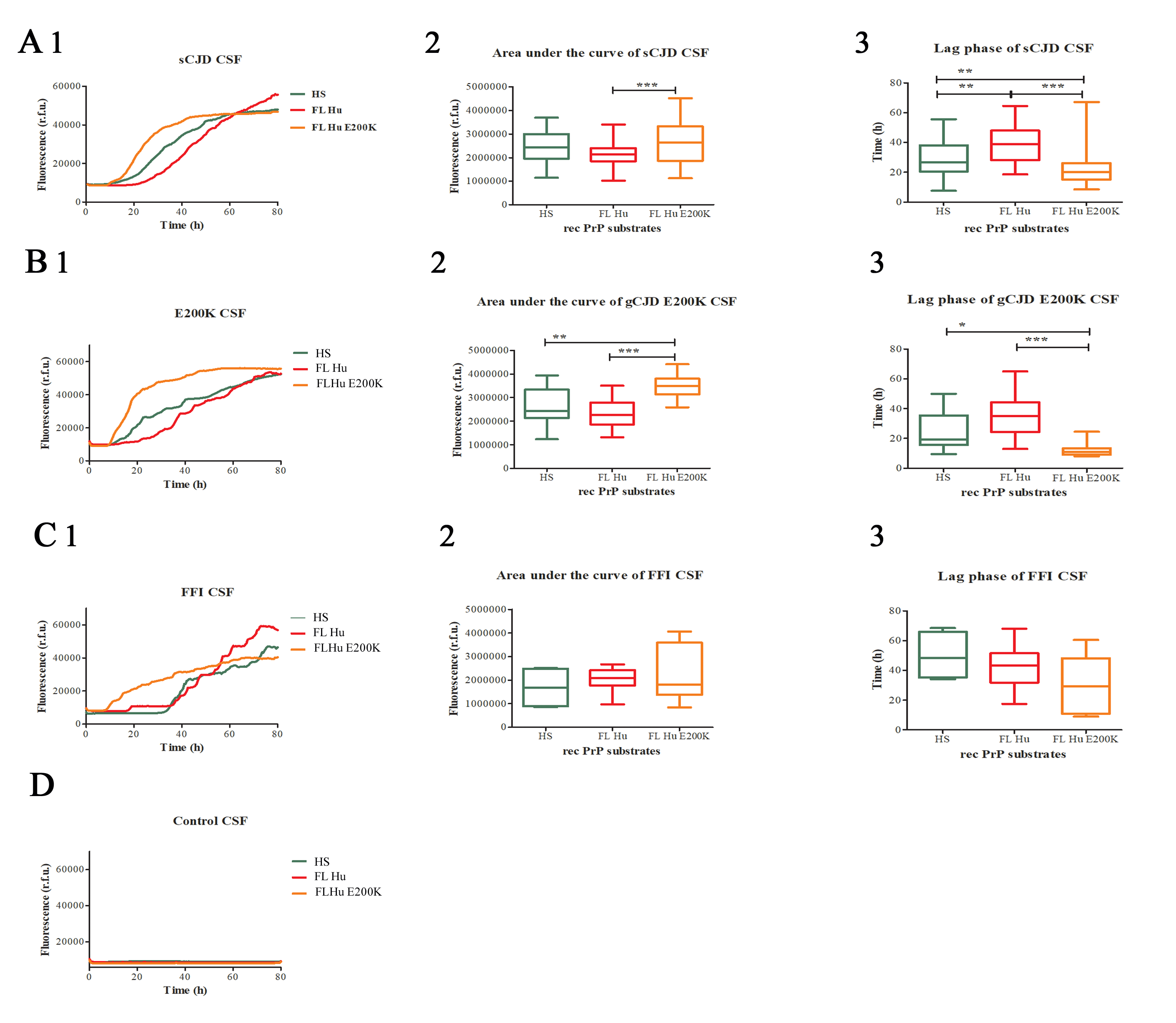

Supplement: Supplementary file 3 — Supplementary Figure 3: RT-QuIC assays seeded with CSF samples from sCJD, gCJD E200K, and FFI patients were used to compare the seeding conversion efficiencies of hamster–sheep (HS), FL Hu, and FL Hu E200K rec PrP substrates. Positive signal response curves of sCJD samples (n = 43) (A1), gCJD E200K samples (n = 16) (B1), and FFI patient samples (n = 22) (C1) are shown for each rec PrP substrate (chimeric HS, FL Hu, and FL Hu E200K). The area under the curve (AUC) (A2, B2, C2) and the lag phase (A3, B3, C3) were used for quantification. For sCJD and gCJD, the rec PrP FL Hu E200K substrate showed the highest seeding conversion efficiency. No seeding conversion was observed in CSF samples from control subjects (n = 44) (D). [file 40478_2025_2212_MOESM3_ESM.tif]

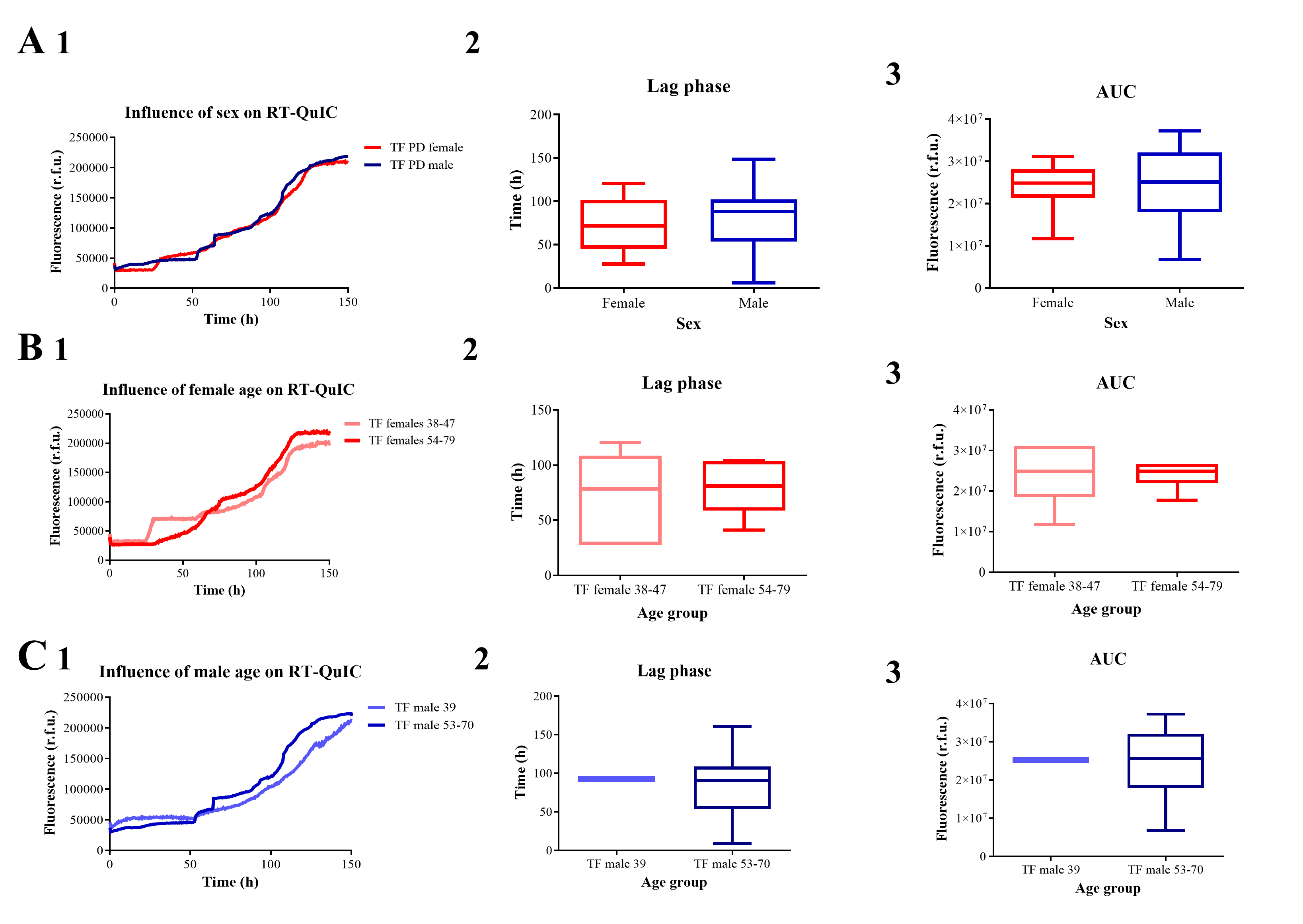

Supplement: Supplementary file 4 — Supplementary Figure 4: Impact of age and gender on the TF RT-QuIC signal response. The kinetic curves of positive TF RT-QuIC reactions from male and female individuals with prion diseases (PD) were compared across different age groups. Mean fluorescence values from TF RT-QuIC assays were analyzed in female (n = 11; sCJD = 7, FFI = 2, GSS = 1, fCJD [T183A] = 1) and male (n = 11; sCJD = 9, GSS = 1, FFI = 1) prion disease samples (A1). Females were further categorized into younger (<50 years, n = 4; sCJD = 3, fCJD [T183A] = 1) and older (>50 years, n = 6; sCJD = 6) groups (B1). Males were similarly grouped into younger (39 years, GSS = 1) and older (53–70 years, sCJD = 7, GSS = 1, FFI = 1) groups (C1). The analysis focused on lag phase duration (2) and area under the curve (AUC) values (3). Neither sex (A1–3) nor age group (B1–3, C1–3) showed significant differences in TF RT-QuIC signal responses. [file 40478_2025_2212_MOESM4_ESM.tif]

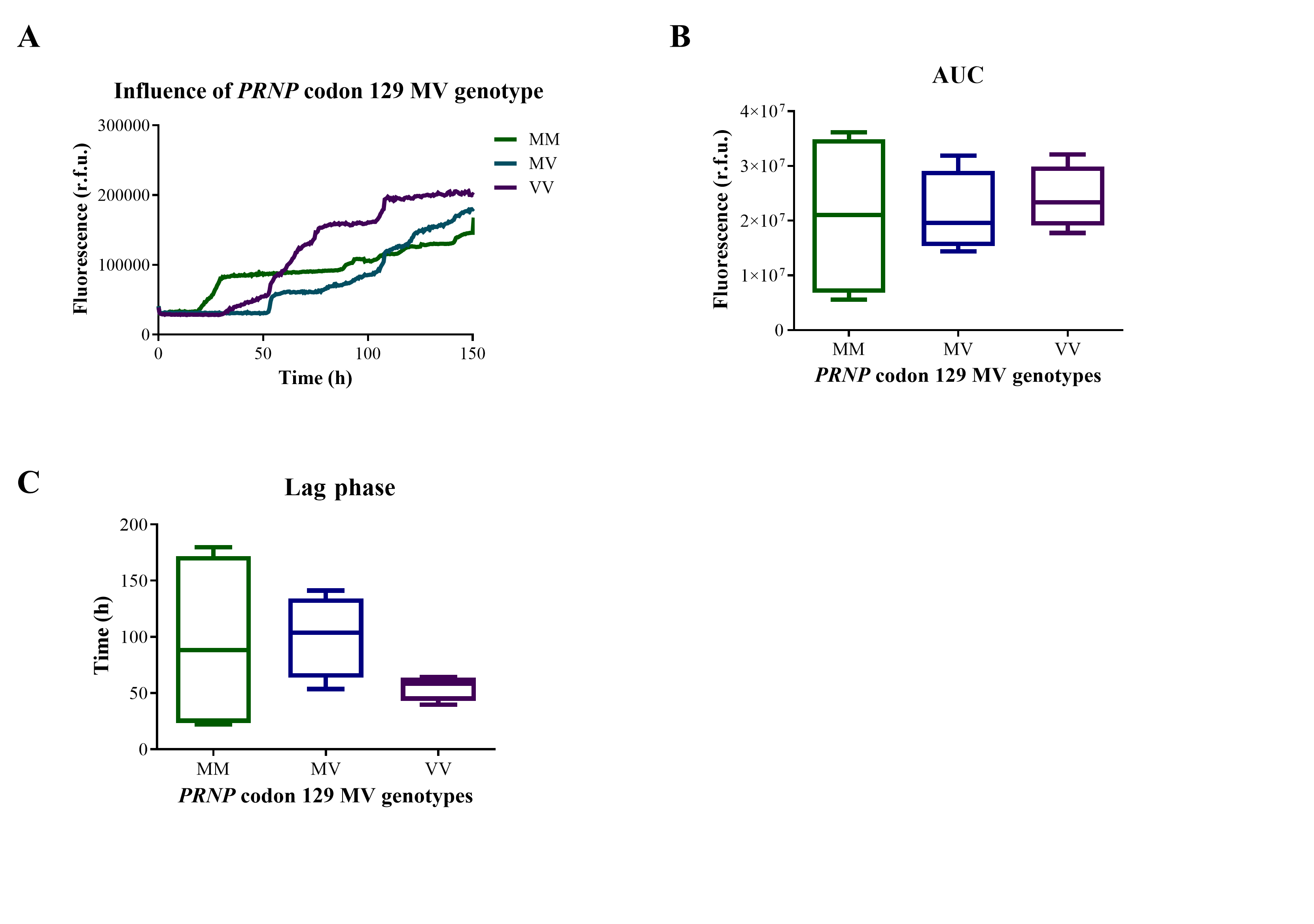

Supplement: Supplementary file 5 — Supplementary Figure 5: Impact of the PRNP codon 129 MV genotype in sCJD on the TF RT-QuIC signal response. A) Comparison of kinetic curves of positive TF RT-QuIC reactions of different PRNP codon 129 MV genotypes in sCJD (MM, MV, VV). B-C) Applying quantitative parameters, such as the area under the curve (AUC) and duration of the lag-phase we observed no significant differences in signal response in the TF RT-QuIC assay among different PRNP codon 129 MV genotypes (n = 4 per group) [file 40478_2025_2212_MOESM5_ESM.tif]

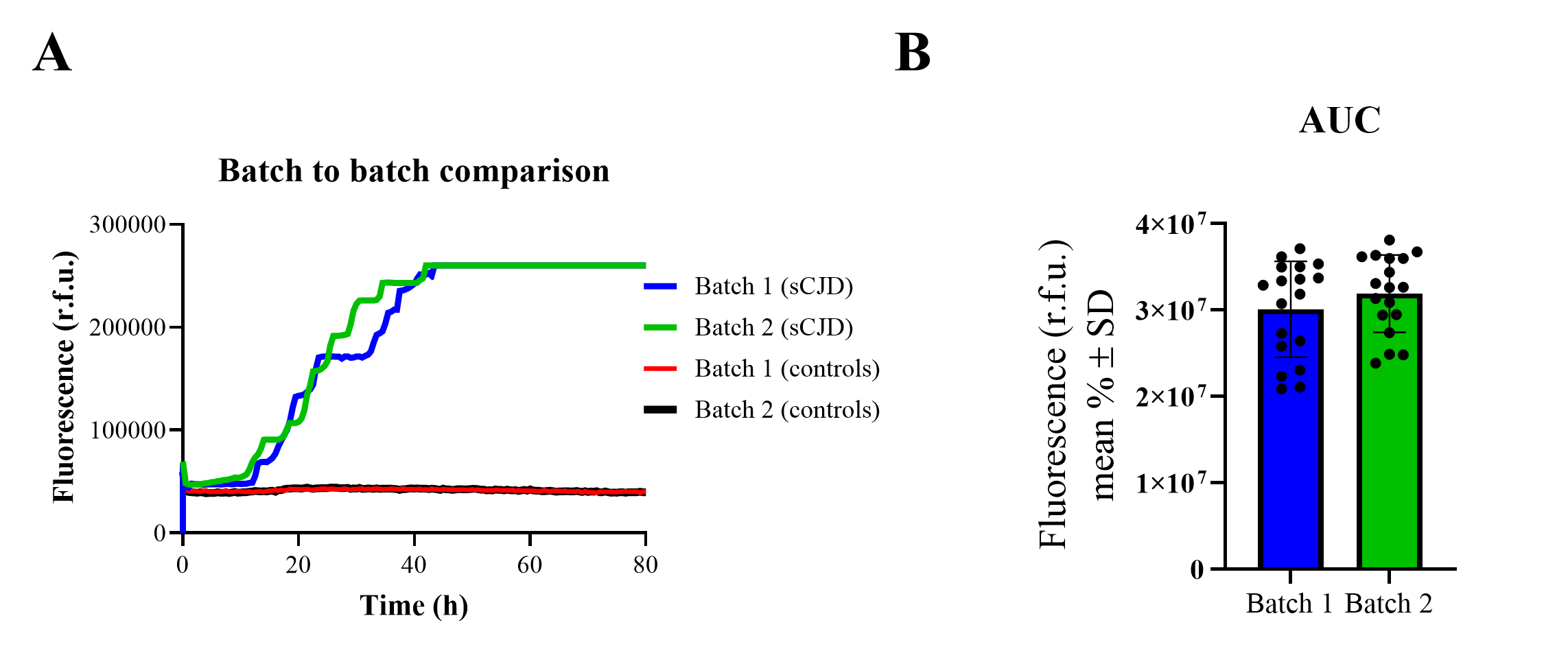

Supplement: Supplementary file 6 — Supplementary Figure 6: RT-QuIC seeding kinetics showing batch-to-batch comparison of recombinant FL Hu E200K substrate. A) Signal responses, seeded with CSF from 6 CJD cases (each 3/3 positive) and 6 non-prion controls (each 0/3 negative), using FL Hu E200K substrate from both batch 1 and batch 2, reveal almost identical kinetic curves. B) Calculation of the area under the curve (AUC) values of sCJD seeded reactions indicated no significant differences between the two batches [file 40478_2025_2212_MOESM6_ESM.tif]

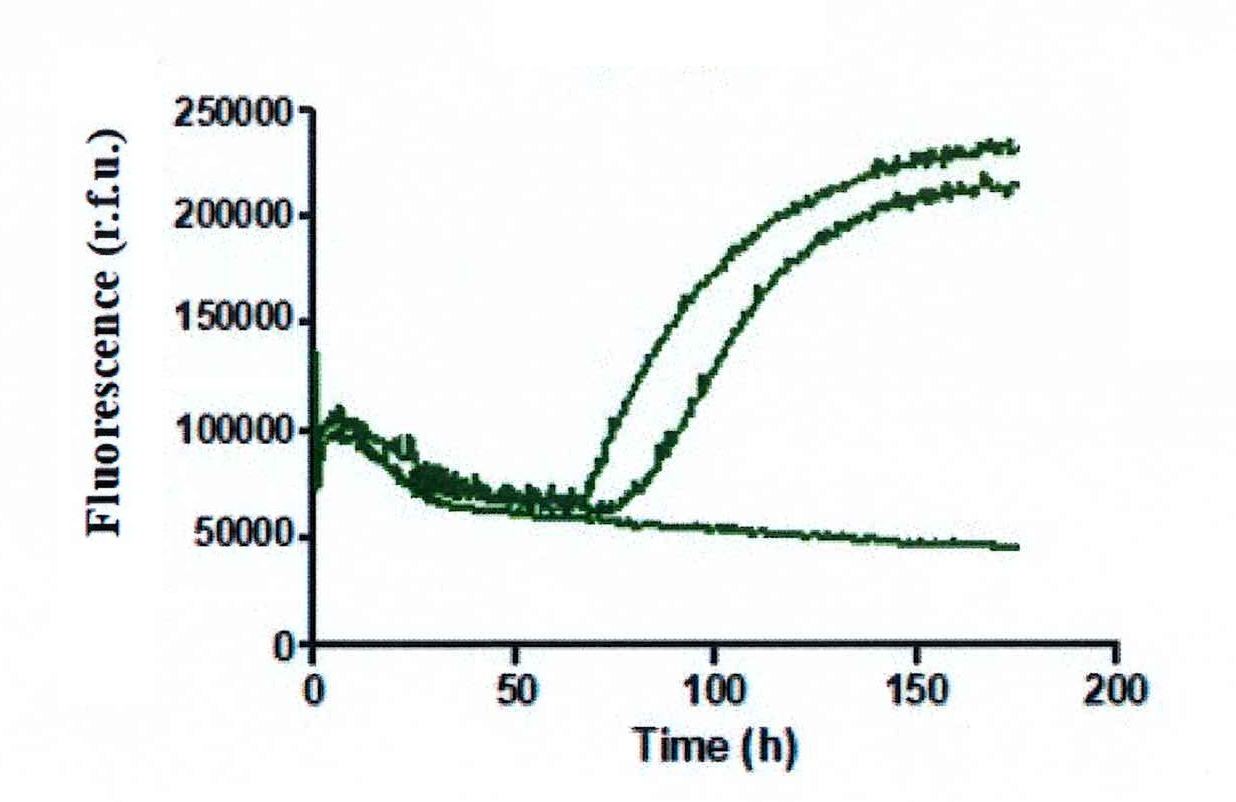

Supplement: Supplementary file 7 — Supplementary Figure 7: RT-QuIC seeding kinetics of a false positive AD-patient. The RT-QuIC was seeded with TF from a non-prion control. Two out of three reactions were considered as positive. The clinical assessment supports a diagnosis of mild to moderate Alzheimer’s dementia [file 40478_2025_2212_MOESM7_ESM.tif]

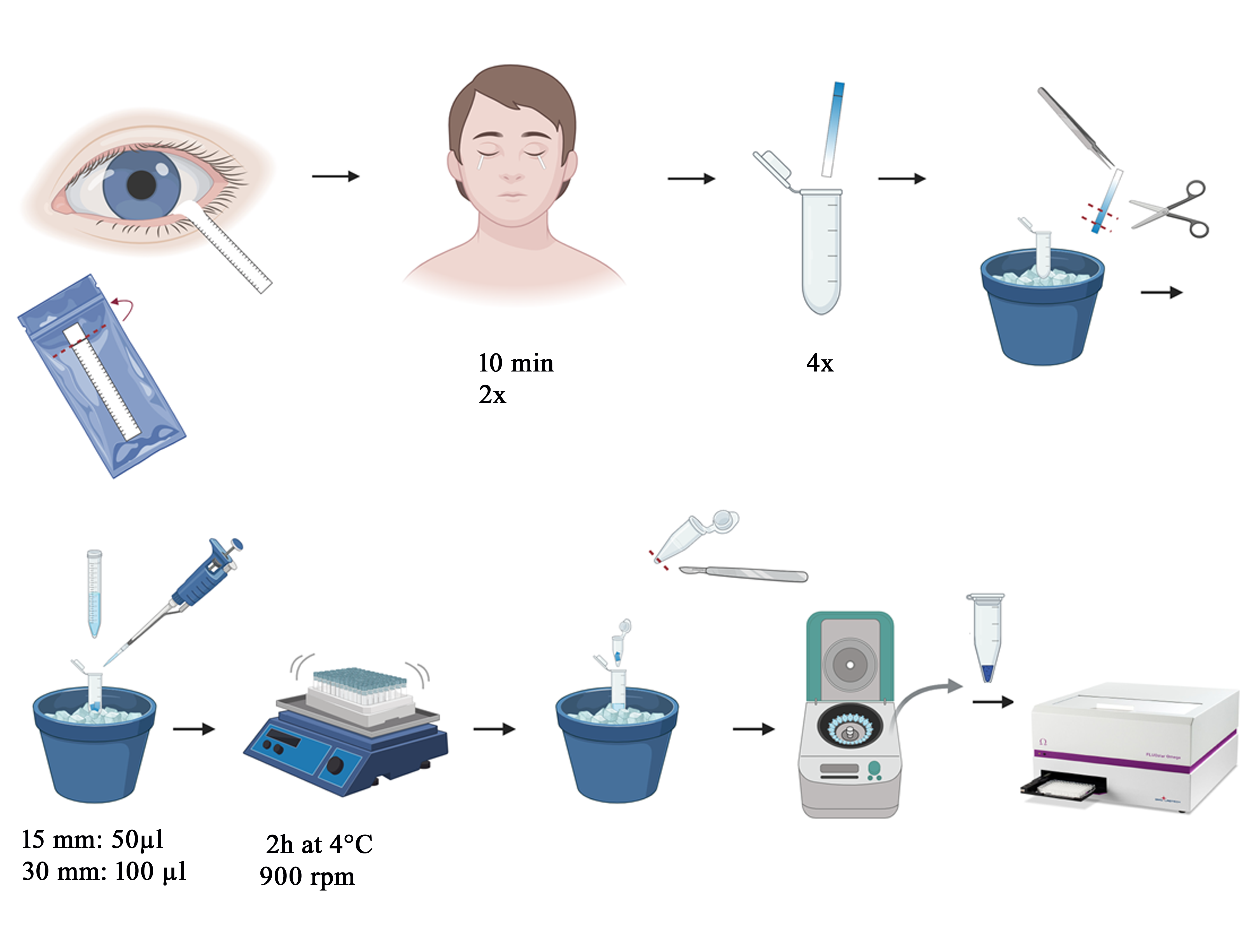

Supplement: Supplementary file 8 — Supplementary Figure 8: Procedure of tear fluid collection and strip extraction. A 5 × 35 mm strip was placed on the outer corner of the lower eyelid for 8–10 min, following the standard Schirmer test for tear production. Samples were frozen within 30 min and stored at − 80 °C without further treatment for storage. For protein isolation, samples were eluted by cutting at least 15 mm of the strip into 5 mm pieces, placing them in a 1.5 mL tube with 100 μL RT-QuIC buffer, vortexing for 1 min, and incubating for 30 min. Strips were then transferred to perforated 0.5 mL tubes placed above the 1.5 mL tubes and centrifuged at 14,000 rpm (Eppendorf centrifuge 5810 R), 4 °C for 30 min to collect the extract. Samples from both eyes were pooled for analysis. [file 40478_2025_2212_MOESM8_ESM.tif]
