# Supplementary material for: Advancing prion diagnostics: full-length human E200K RT-QuIC substrate facilitates prion detection in tear fluid and improves sensitivity in cerebrospinal fluid
Source: Acta Neuropathol Commun. 2026 Jan 22;14:28. doi: 10.1186/s40478-025-02212-8 (PMC12849081; doi:10.1186/s40478-025-02212-8)
Supplement: Supplementary file 9 — Supplementary Material 9 [file 40478_2025_2212_MOESM9_ESM.docx]

**Supplementary Appendix**

**Production of recombinant PrP substrates**

**Bacteria culture and protein expression**

A single colony from an overnight LB agar plate was picked and added to the 4 ml LB medium with 50 μg/ml kanamycin (pET41a(+) vector is resistant to kanamycin) or 100 μg/ml ampicillin (pET-11a vector is resistant to ampicillin) with 34 μg/ml chloramphenicol in a 50 ml Falcon tube. Two mini cultures were prepared and incubated at 37 °C, 250 rpm for 6 hours. For protein overexpression**, an Overnight Express Autoinduction System 1 (Sigma Aldrich, USA) was added** to 1 L of LB medium. The cells were then shaken at 250 rpm at 37 °C for 20 h, centrifuged (10000 x g for 10 min), and the supernatant discarded. The pellet was **weighed** (±16 g per 1 L) and stored in 50 ml Falcon tubes (4 Falcon tubes with ±4 g each) at -20 °C overnight or -80 °C until further use.

**Inclusion Bodies Purification**

The inclusion bodies were purified using BugBuster Mix (Merck Millipore, USA) according to the manufacturer’s instructions with slight modifications. Each of the 4 Falcon tubes containing ±4g of bacteria pellet was placed on ice to thaw for 30 min before being resuspended by pipetting up and down and homogenized (Bandelin Sonopuls, Germany) with 14 ml room temperature of 1X Bug Buster Master Mix (Merck Millipore, USA) for 1 min, 5 cycles at 45 % power. Each bacteria cell suspension was incubated on a rotating mixer at room temperature for 20 min, followed by centrifugation at 13,000 g for 20 minutes at 4 °C. The supernatant was discarded, and the bacteria pellet resuspended in 14 ml of 1X Bug Buster Master Mix using the same pipette as before and re-homogenized (1 min, 5 cycles at 45 % power) before incubation at room temperature for 20 min. After 20 min, 0.1X Bug Buster Master Mix (Merck Millipore, USA) was added to the cell suspension until it reached the total volume of 40 ml before centrifugation (13,000g, 15 min at 4 °C). Afterwards, the supernatant was discarded. The pellet was resuspended by pipetting up and down with 30 ml room temperature of 0.1X Bug Buster Master Mix (Merck Millipore, USA) before centrifugation (8,000g, 15 min at 4°C). After centrifugation, the supernatant was discarded, and the inclusion body pellet was stored at -20°C overnight.

**Protein Purification**

Each inclusion body pellet in 50 ml Falcon tubes from the pellet of 1L bacterial culture (total of 4 Falcon tubes per 1L of bacteria culture) was placed on ice to thaw for 30 min before being dissolved by adding 14 ml of 8M guanidine (38g guanidine in 0.1M NaPO_4_ at pH 8) and homogenized (Bandelin Sonopuls, Germany) for 1 min 5 cycles at 45% power. Dissolved inclusion bodies suspension was incubated on a rotating mixer at room temperature for 50 min before centrifugation (13,000g, 5 min at 4 °C). After centrifugation, each supernatant was mixed with equilibrated 18g of Ni-NTA beads in 30 ml Denaturing buffer (6M GdnHCl in 0.1M NaPO_4_ at pH 8) for 40 min to allow the proteins to bind to the beads. After incubation, 3X 18 g incubated resin was pulled and loaded into the Akta #XK26 column, and 1X 18 g incubated resin was loaded into Akta #XK16 column. The column was attached to FPLC, and the following gradient refolding program was run: 100% A (Denaturing Buffer) and 0% B (Refolding Buffer (0.1M NaPO_4_ at pH 8)) to 0% A and 100% B for 240 min (0.75 ml/min for #XK16 column with 18g beads and 2.20 ml/min Akta #XK26 column with 3X 18g beads). After gradient refolding an additional 30 min of 100% B (Refolding Buffer) was allowed to flow through the column. To elute the protein, a gradient elution program was run: 100% A (Refolding Buffer) and 0% B Elution Buffer (0.5M C_3_H_4_N_2_ in 0.1M NaPO_4_ at pH 5.8) to 0% A and 100% B for 50 min (2 ml/min for #XK16 column with 18 g beads and 6 ml/min Akta #XK26 column with 3X 18g beads). The fraction tubes with the eluted proteins were collected from the center of the large UV 280 peak and combined before being diluted with 1/3 volume of Dialysis Buffer at 4 °C (10mM NaPO_4_ at pH 5.8). The diluted protein was filtered through a sterile disposable 0.22 μm filter (Merck Millipore, USA) before being placed into a SnakeSkin dialysis tube with a 10K molecular weight cut-off (Thermo Fisher, USA) for dialysis in 3.6 L of dialysis buffer at 4°C for 1–4 hours, then transferred to fresh 3.6 L dialysis buffer for overnight incubation at 4°C.

After overnight incubation, the SnakeSkin tubing was transferred into a fresh 3.6L dialysis buffer for 4 h before the proteins were filtered once again with a sterile disposable 0.22 μm filter (Merck Millipore, USA) and the 2 batches combined (1X 18 g beads and 3X 18 g beads). The protein concentration was measured using NanoDrop 1000 UV Visible Spectrophotometer (Thermo Scientific, USA) at 280 nm and samples were stored at -80°C.

The protein concentration was estimated by NanoDrop 1000 UV visible Spectrophotometer (Thermofisher, USA). Protein A280 application was selected from the main menu of the NanoDrop software, and 2 μl of the dialysis buffer (10 mM Sodium Phosphate, pH 5.8) was pipetted into the bottom pedestal for blank measurement. The blank solution (dialysis buffer) was wiped from the lower and upper pedestals, and 2 μL of the purified protein sample was loaded onto the bottom pedestal, and the concentration was measured. The purified protein sample was wiped from the upper and lower pedestals using a dry laboratory wipe, and 2 μL ddH_2_O was added and wiped again before the subsequent sample measurement.
