# Supplementary material for: Advancing prion diagnostics: full-length human E200K RT-QuIC substrate facilitates prion detection in tear fluid and improves sensitivity in cerebrospinal fluid
Source: Acta Neuropathol Commun. 2026 Jan 22;14:28. doi: 10.1186/s40478-025-02212-8 (PMC12849081; doi:10.1186/s40478-025-02212-8)
Supplement: Supplementary file 10 — Supplementary Material 10 [file 40478_2025_2212_MOESM10_ESM.docx]

**Table S1. Demographic characteristics of the tear fluid patient cohort.**

|  | **Sex (f/m)** | **Age median (min-max)** | **Disease duration in months median (min-max)** | ***PRNP* Codon 129 MV genotype** |
| --- | --- | --- | --- | --- |
| **Total CJD** | **12/22** | **66 (35-85)** | **8 (1-27) (n=26)*** | **14 MM, 8 MV, 5VV *** |
| Definitive sCJD | 2/3 | 68 (62-76) | 12 (2-27) | 4MM* |
| Probable sCJD | 10/19 | 65 (35-85) | 4 (1-17) (n=23)* | 10 MM, 8 MV, 5VV* |
| **Genetic prion diseases** | **5/6** | **44 (32-57)** | **29 (10-69)** | **6MM, 4MV*** |
| FFI | 0/5 | 49 (32-57) | 12 (10-13) (n=3)* | 5MM |
| GSS | 3/1 | 43 (39-46) | 29 (n=1)* | 1 MM, 2 MV* |
| T183A | 2/0 | 41 (39-43) | 46 (24-69) | 2 MV |
| **HMC^**^** | **19/9** | **47 (32-73)** | **-** | **14 MM, 2MV, 3VV** |
| FFI | 9/1 | 44 (32-65) | - | 8 MM, 2MV |
| 5-OPRI | 3/1 | 60 (54-64) | - | 3 VV* |
| GSS | 1/4 | 39 (39-41) | - | 4 MM* |
| E200K | 5/3 | 58 (44-73) | - | 1 MM* |
| G114V | 1/0 | 36 | - | 1 MM |
| **Non-prion diseases** | **54/40*2** | **55 (23-43)*** | **-** | **-** |

^*^ Rest is unknown

^**^ HMC = healthy mutation carriers (non-symptomatic persons at risk)
