# Supplementary material for: Advancing prion diagnostics: full-length human E200K RT-QuIC substrate facilitates prion detection in tear fluid and improves sensitivity in cerebrospinal fluid
Source: Acta Neuropathol Commun. 2026 Jan 22;14:28. doi: 10.1186/s40478-025-02212-8 (PMC12849081; doi:10.1186/s40478-025-02212-8)
Supplement: Supplementary file 11 — Supplementary Material 11 [file 40478_2025_2212_MOESM11_ESM.docx]

**Table S2. List of recommendation for a reliable application of TF RT-QuIC.**

| **Parameter** | **Recommendation** |
| --- | --- |
| Amount of TF on the Strip per reaction | 15-20 mm (>10 µL) |
| Duration of the TF RT-QuIC | 150h |
| Software settings of the TF-QuIC | 1 min double orbital shaking at 700 rpm and 1 min incubation |
| Definition of a positive reaction | ≥ 50% positive reactions |
| 1out of positive reactions | Repetition, if 3/6 are positive reaction is considered positive |
| Kind of recombinant substrate | Highly sensitive FL Hu E200K |
| Collection of TF | Schirma method |
